# EcoNet-Multi: optimising deep learning frameworks for robust waste detection in diverse global environments

**DOI:** 10.3389/fpls.2026.1878199

**Published:** 2026-07-28

**Authors:** Alok Kumar Shukla, Shubhra Dwivedi

**Affiliations:** Thapar Institute of Engineering and Technology, Patiala, Punjab, India

**Keywords:** deep learning, environmental, optimisation, recycling, surrogate model, waste classification

## Abstract

In the current era of technological advancement, the increasing demand for automated environmental monitoring and smart recycling systems plays a crucial role in maintaining a healthy environment for both humans and nature. Deep learning and artificial intelligence-based waste classification have attracted significant attention for effective waste segregation. In the existing literature, several deep learning algorithms are available for image classification in waste management systems. However, hyperparameter optimisation remains a dual challenge: maximising classification accuracy whilst preserving balanced class detection across highly imbalanced waste categories. Addressing these challenges, this paper proposes a Surrogate-Assisted Hybrid Multi-Objective Binary Bat with Cuckoo Search algorithm, termed SMOBBCS (Multi-Objective Binary Bat-Cuckoo Search), which simultaneously minimises classification error and the complement of the macro-F1 score for waste category detection. The algorithm fuses the inertia-weighted frequency-modulated velocity update of the Binary Bat Algorithm with the Lévy-flight perturbation mechanism of Cuckoo Search, governed by a smooth adaptive transition gate that facilitates population evolution from global exploration to local exploitation. A lightweight *k*-NN surrogate model reduces the number of true fitness evaluations without sacrificing solution quality. Experimental results validate the proposed approach against five state-of-the-art surrogate-assisted multi-objective optimisers (SMEPO, SMWOA, SMBCS, SMPSO, and SMBAT) across four real-world waste detection benchmarks: HGCD, TrashNet, WasteNet, and Realwaste. Four deep learning classifiers (CNN, DNN, GRU, and CNN-DNN) are employed for comprehensive evaluation. The proposed SMOBBCS method achieves mean accuracy of 92.71%, mean Macro-F1 score of 0.9209, mean precision of 0.9225, mean recall of 0.9205, and mean Matthews Correlation Coefficient (MCC) of 0.9052, demonstrating superior performance in automated waste classification tasks.

## 1 Introduction

### 1.1 Background and context

The global waste management crisis presents one of the most pressing environmental challenges of the 21st century (Ibitoye et al., 2026). According to the World Bank report, global waste generation is projected to increase by 70% from 2016 levels, reaching 3.4 billion tonnes annually by 2050 (Mustafa et al., 2026; Narang et al., 2026). This exponential growth, driven by urbanisation, population expansion, and changing consumption patterns, overwhelms traditional waste management infrastructure and contributes significantly to environmental degradation, greenhouse gas emissions, and public health hazards. As a result, global increases in urbanisation and consumption have placed unprecedented strain on waste management systems, resulting in environmental degradation, resource depletion, and public health issues. Efficient waste management therefore plays a crucial role, as growing populations and evolving consumer preferences continue to challenge the resilience of our ecosystems (Ghosh and Goswami, 2026).

In the literature, various approaches for the detection and classification of solid waste have been explored, including both traditional and object detection methods (Dwivedi and Shukla, 2025; Srivastava et al., 2025). However, traditional manual sorting operations face multiple constraints: labour costs constituting 50–70% of facility operational expenses; human sorters processing only 30–60 items per minute with declining accuracy; significant health and safety risks; and classification inconsistency varying with operator fatigue and material complexity (Khonjun et al., 2026). Consequently, automated waste classification powered by AI and deep learning offers transformative potential, with recent CNNs demonstrating accuracies exceeding 90% on controlled datasets (Thanjaivadivel and Rajasekaran, 2026). Nevertheless, the transition to robust production-ready systems faces critical challenges related to hyperparameter sensitivity, multi-objective trade-offs, dataset diversity, and computational constraints.

Machine learning approaches have emerged as powerful tools for waste detection and classification. Image processing and computer vision techniques, combined with machine learning algorithms, enable automated waste detection and classification based on visual characteristics. Given the severity of class imbalance in real-world waste streams, hyperparameter optimisation (HPO) for waste classification constitutes an inherently multi-objective problem: practitioners seek to simultaneously *minimise* classification error and *maximise* macro-F1 score, the latter explicitly addressing imbalance where categories such as hazardous medical waste may constitute fewer than 2% of collected items (Liu et al., 2026). These criteria partially conflict aggressively decreasing learning rates can degrade minority-class recall making multi-objective evolutionary optimisation (MOEO) a natural framework.

Considering the large number of target categories in existing research, most evolutionary optimisation models may not efficiently accomplish this task (Tank and Donga, 2026). However, metaheuristic algorithms provide effective solutions for deep learning HPO, offering robust optimisation over non-convex, discrete-continuous mixed spaces (Krishnan et al., 2026; Shukla et al., 2026). Among these approaches, the Bat Algorithm (BA) (Yang, 2010) and Cuckoo Search (CS) (Yang and Deb, 2009) have distinguished themselves through simplicity and effectiveness: BA achieves competitive exploitation via frequency-modulated echolocation, whilst CS maintains diversity through Lévyflight global jumps, whose heavy-tailed step-length distribution underpins efficient random-search behaviour (Viswanathan et al., 1999). Prior hybridisation studies, predominantly single-objective, confirm that BA–CS combinations improve convergence speed and solution diversity (Zhang et al., 2024). Nevertheless, premature convergence in high-dimensional spaces and insufficient Pareto-front diversity on imbalanced benchmarks remain open challenges.

### 1.2 Research contributions

This paper makes the following contributions to waste classification and multi-objective hyperparameter optimisation:

1. Hybrid Multi-Objective Algorithm: SMOBBCS combines the frequency-modulated exploitation of Binary Bat Algorithm with the Lévy-flight global exploration of Cuckoo Search through a smooth time-varying adaptive gate, explicitly designed for bi-objective hyperparameter optimisation balancing classification accuracy and macro-F1 score on highly imbalanced waste datasets.
2. Surrogate-Assisted Evaluation: A lightweight *k*-NN surrogate model (*k* = 5) reduces true deep learning fitness evaluations by 65% per iteration, achieving mean wall-clock runtime of 829.6 seconds per dataset whilst maintaining Pareto archive quality.
3. Comprehensive Experimental Validation: Rigorous evaluation across four real-world waste benchmarks (HGCD: 12 classes, 15,000 images; TrashNet: 6 classes, 2,527 images; WasteNet: 5 classes, 2,000 images; Realwaste: 9 classes, 4,752 images) using four deep learning architectures (CNN, DNN, GRU, CNN-DNN), demonstrating consistent superiority over five state-of-the-art surrogate-assisted multi-objective algorithms (SMEPO, SMWOA, SMBCS, SMPSO, SMBAT).
4. Quantitative Performance Gains: SMOBBCS achieves mean accuracy of 92.71%, mean Macro-F1 of 0.9209, mean MCC of 0.9052, demonstrating consistent gains across balanced and imbalanced datasets.

### 1.3 Paper organisation

The remainder of this paper is structured as follows. Section 2 reviews related work on waste classification, hyperparameter optimisation, and multi-objective evolutionary algorithms. Section 3 describes the deep learning architectures (CNN, DNN, GRU, and CNN-DNN) employed for waste classification. Section 5 describes the experimental setup and presents comprehensive results across all four benchmarks together with complexity and limitation analyses. Finally, Section 6 summarises the findings and their implications for sustainable waste management.

## 2 Related work

This section surveys prior work on waste classification and hyperparameter optimisation. Specifically, the review is organised into four thematic strands: deep learning approaches for waste detection; hyperparameter optimisation methods; multi-objective formulations; and nature-inspired meta-heuristics (Islam et al., 2025).

### 2.1 Waste classification using deep learning

The application of deep learning to waste classification has evolved rapidly over the past decade. For instance, robust waste image classification and object detection have been studied across 28 different recyclable categories totalling 10,406 images. For the waste classification task (Sayem et al., 2025), proposed a novel dual-stream network that outperforms several state-of-the-art models with an overall classification accuracy of 83.11%. The same study also introduced the GELAN-E (generalised efficient layer aggregation network) model to solve the waste object detection problem, obtaining a mAP50 of 63%, which surpassed other state-of-the-art detection models. These advances mark a significant leap forwards in the arena of intelligent waste management and pave the way for more efficient and effective solutions.

This line of research aims to provide an intelligent IoT-based garbage bin system, with classification performed using deep learning techniques; such smart bins can detect a wider range of garbage types directly in the home. Although IoT and machine learning have produced several technical success stories, there remains a need for sustainable, environmentally friendly technologies to manage daily waste. The IoT-based garbage system in (Song et al., 2024) uses several sensors – humidity, temperature, gas, and liquid – to identify the condition of the garbage. A Smart Garbage Bin system is first designed, and data are then collected through a garbage-annotation application; a deep learning method is subsequently used for garbage image detection and classification. Object detection is performed using Improved RefineDet (IRD) with an Arithmetic Optimisation Algorithm (AOA), and the EfficientNet-B0 model is used to classify the detected garbage images. The trained deep learning model is evaluated on smart bins in real time, with region-specific litter photographs used to fine-tune and improve classification accuracy.

The proposal is to implement a Smart Dustbin System (SDS) in congested urban areas for maintaining hygiene. This paper presents a comprehensive analysis of smart dustbin system applications after a detailed literature review and discussion of the recent research which is expected to help in improving the waste management systems (Arthur et al., 2024). The real-time SDS in use is up-to-date with the latest developments in deep learning, computer vision and the Internet of Things. The smart dustbin system used in day-to-day life reduces the overloading of bins, minimises labour cost and saves energy and time. It also helps keep cities clean, reducing the chance of disease transfer. Universities, shopping malls and high-rise buildings are the primary users of the SDS. The evolution of the SDS through the years with different features and technologies is well analysed. AI perception provides the datasets used for Smart Waste Management and benchmark garbage image datasets. The potential limitations of these works are demonstrated by comparing the results of the existing works.

The assessment allows us to identify the strengths and weaknesses of these models and choose the most suitable one for a given task (Goswami et al., 2024). This indicates a major challenge in the lack of recent comparative studies of object detection models across different image qualities, object sizes, and training data scales. The above challenges are addressed by a rigorous evaluation of state-of-the-art object detection models including YOLO-v8, Faster R-CNN with ResNet backbones, and End-to-End Object Detection Transformers (DETR) through a comprehensive assessment framework comprising mean Average Precision (mAP), accuracy, and inference speed across diverse waste classification datasets.

The work in (Poudel and Poudyal, 2022) has as its main objective the classification of waste material images into seven categories (cardboard, glass, metal, organic, paper, plastic, and trash) using a CNN, with the cardboard, organic, and paper classes treated as biodegradable waste and the remaining classes treated as non-biodegradable waste. Several pre-trained CNN models – InceptionV3, InceptionResNetV2, Xception, VGG19, MobileNet, ResNet50, and DenseNet201 – were fine-tuned on the waste dataset; amongst these, VGG19 achieved the lowest accuracy whilst InceptionV3 achieved the highest. The results obtained were, in general, encouraging.

(Lu et al., 2020) proposed a smart waste classification and collection system (SWCCS) based on ICT, abstracted as a bi-objective mathematical programming model for optimising the waste-collection problem. To implement the proposed SWCCS efficiently, the authors designed a novel multi-objective hybrid algorithm combining whale optimisation and genetic algorithms (MOGWOA) with an improved convergence factor and a fast non-dominated sorting method. Experimental results comparing MOGWOA with two classical multi-objective algorithms, on both generated test instances and a real-world case, demonstrate that MOGWOA optimises the established model more effectively. The paper describes the operation of the ICT-based SWCCS and how it can help sanitation companies collect waste more efficiently and sustainably.

In order to determine the best approach, the study in (Do and Phan, 2026) offered a thorough comparison of three paradigms: machine learning algorithms that use handcrafted features; deep learning architectures, such as ResNet variants, EfficientNetV2S, DenseNet121, and MobileNetV3; and a hybrid approach that combines different ML classifiers with deep models for feature extraction. Experiments on three public datasets show that the hybrid approach regularly performs better than the others, surpassing state-of-the-art benchmarks with accuracy of up to 99.72% on TrashNet, 100% on Household, and 99.87% on Garbage. Additionally, feature selection speeds up training and inference by reducing feature dimensionality by more than 95% without sacrificing accuracy. In addition to establishing more trustworthy standards, this study presents an effective hybrid architecture that achieves high accuracy whilst lowering inference costs, making it appropriate for scalable implementation. Collectively, prior hybridisation studies confirm that BA–CS combinations improve convergence speed and diversity. However, to the best of our knowledge, SMOBBCS is the first multi-objective, surrogate-assisted formulation of this hybrid specifically designed for the waste classification domain.

## 3 Deep learning architectures for waste classification

### 3.1 Convolutional neural network

Convolutional Neural Networks (CNNs) constitute a pivotal class of deep learning architectures that employ spatially-shared convolutional filters to extract hierarchical feature representations from image data (Thanjaivadivel and Rajasekaran, 2026). Within the waste classification context, one-dimensional projected feature maps from the ResNet18 backbone are processed by CNN heads that detect local statistical correlations without manual feature engineering. The architecture alternates between convolutional layers which apply learned filter banks to produce feature maps and pooling layers that progressively reduce spatial resolution whilst retaining salient discriminative information. A fully connected classification head maps extracted representations to waste-class probabilities. The convolution operation is formalised as:

(1) $$z_{i}^{b}=\sigma [\sum_{j\in l_{i}}z_{j}^{b-1}\;w_{ji}^{b}+d_{j}^{b}]$$

where *σ* is the activation function, *l_i_* is the input attribute set of layer (*b*−1), $w_{ji}^{b}$ is the connection weight between node *j* of layer (*b*−1) and node *i* of layer *b*, and $d_{j}^{b}$ is the bias. The pooling layer follows:

(2) $$z_{i}^{b}=\sigma [\alpha_{i}^{b}\cdot \text{csf}\;(z_{i}^{b-1}+d_{i}^{b})]$$

where *α* is the weighting matrix and csf is the sub-sampling function. The fully connected output layer produces:

(3) $$x^{n}=\sigma [w^{n}z^{n-1}+d^{n}]$$

where *x^n^* is the fully connected layer output, *z*^(^*^n^*^−1)^ its input, *w^n^* the weight, and *d^n^* the deviation.

### 3.2 Deep neural network

Deep Neural Networks (DNNs) are feed-forward architectures comprising multiple hidden layers between input and output, enabling modelling of highly non-linear decision boundaries (Jano et al., 2026). In waste classification, DNNs receive ResNet18 feature vectors and learn class-discriminative representations through successive affine transformations followed by non-linear activations. Batch Normalisation stabilises internal covariate shift; Dropout regularisation prevents overfitting when minority waste classes have limited support. The Softmax output converts raw logits to calibrated class posteriors. The hidden layer computation for input [*X*_1_*,X*_2_*,…,X_mn_*] is:

(4) $$h_{i}(x)=f\;(w_{i}^{T}x+b_{i})$$

where *f* is the activation function. The Softmax output layer is:

(5) $$\text{Softmax}(x_{i})=\frac{e^{x_{i}}}{\sum_{j=1}^{J}e^{x_{j}}}$$

ReLU, *F*(*x*) = max(0*,x*), is used for hidden layers to prevent vanishing gradients. The mean loss function is:

(6) $$L(x_{n},y_{n})=\frac{1}{n}\sum_{i=1}^{n}L\;(y_{n},f(x_{n}))$$

### 3.3 Gated Recurrent Unit

Gated Recurrent Units (GRUs) simplify LSTM by combining the input and forget gates into a single update gate *z_t_* and introducing a reset gate *r_t_* (Eryeşil et al., 2026). The Gated Recurrent Unit is a streamlined variant of the Long Short-Term Memory (LSTM) architecture, specifically engineered to alleviate the vanishing gradient problem whilst maintaining a lower computational overhead. By merging the traditional forget and input gates into a single update gate (*z_t_*), the GRU dictates how much of the previous memory should be retained versus how much new information should be integrated. Additionally, the reset gate (*r_t_*) modulates the influence of the past hidden state on the current candidate activation ( ${\tilde{h}}_{t}$), allowing the model to effectively forget irrelevant historical data. This reduction in parameter count achieved by eliminating the separate cell state often results in faster convergence and improved generalisation on smaller or moderate-sized datasets, such as those utilised in this study, without a significant loss in performance compared to its more complex predecessors.

(7) $$z_{t}=\sigma (W_{z}x_{t}+U_{z}h_{t-1})$$

(8) $$r_{t}=\sigma (W_{r}x_{t}+U_{r}h_{t-1})$$

(9) $${\tilde{h}}_{t}=\tanh\;(W_{h}x_{t}+U_{h}(r_{t}⊙h_{t-1}))$$

(10) $$h_{t}=(1-z_{t})⊙h_{t-1}+z_{t}⊙{\tilde{h}}_{t}$$

GRUs train faster than LSTMs with fewer parameters, making them suitable for the moderate-sized waste datasets used in this study.

### 3.4 CNN-DNN hybrid

The CNN-DNN hybrid architecture synergistically combines convolutional feature extraction with deep neural network classification (Nazari et al., 2025). The ResNet18 backbone first produces spatial feature maps $F\in {\mathbb{R}}^{B\times d}$, which are then flattened and fed to a multi-layer DNN classifier. This combination is particularly effective for waste classification as it leverages both local texture patterns (CNN) and global semantic representations (DNN). The hybrid output is formalised as:

(11) y=Softmax (DNN (CNN(x)))

where *x* is the input image and *y* is the predicted class distribution. A detailed description of the deep learning models employed in this study is provided, spanning from Equations 1–12.

## 4 SMOBBCS architecture: multi-objective hyperparameter optimisation

### 4.1 Problem formulation

The hyperparameter optimisation problem is cast as a bi-objective combinatorial optimisation task over the hyperparameter search space, where each dimension corresponds to one of the *d* hyperparameters. Two conflicting objectives are minimised simultaneously: the classification error *f*_1_ (Equation 13) penalises misclassification across all waste classes; and the complement of the macro-F1 score *f*_2_ (Equation 14) explicitly penalises poor minority-class recall, a critical consideration for imbalanced waste datasets. The bi-objective Pareto framework returns a diverse, non-dominated archive of solutions rather than a single point, allowing practitioners to select an operating point that matches their deployment constraints.

Let *θ* denote a hyperparameter configuration vector over the search space. The bi-objective optimisation problem is:

(12) $$\underset{\theta }{\min}\;F(\theta )={\left[f_{1}(\theta ),\;f_{2}(\theta )\right]}^{⊤}$$

Where

(13) $$f_{1}(\theta )=1-\text{Acc}(\theta )$$

(14) $$f_{2}(\theta )=1-\text{F}1_{\text{macro}}(\theta )$$

and a scalar surrogate fitness combining both objectives:

(15) $$\phi (\theta )=w_{1}f_{1}+w_{2}f_{2},\;w_{1}=0.60,\;w_{2}=0.40$$

Diverse initialisation is essential for multi-objective optimisers because the quality of the initial population directly determines the breadth of the Pareto front discovered during the run. Each of the *N_p_* agents is represented as a *d*-dimensional binary vector drawn uniformly at random, ensuring that all regions of the feature-selection hypercube have nonzero probability of being covered at the start. The continuous velocity matrix **V** is initialised with small random values drawn from $U{\left[-1,1\right]}^{N_{p}\times d}$, providing nontrivial initial frequency-update magnitudes. The Pareto archive is seeded as empty so that only genuinely non-dominated solutions accumulate during optimisation.

The Binary Bat Algorithm (BBA) component of SMOBBCS drives the structured exploitation phase of the search by emulating echolocation through frequency-modulated velocity updates. Each agent adjusts its flight velocity according to a randomly drawn echolocation frequency *f_i_* that scales the displacement towards the current globally best non-dominated solution **x**^∗^. A sigmoid transfer function maps the resulting continuous velocity to a probabilistic bit-flip probability, allowing meaningful exploration of the binary feature space. The inertia weight *w*(*t*) decays linearly from *w*_max_ to *w*_min_ over the run, gradually shifting the population from broad global exploration early in the search towards refined local exploitation as the archive matures. A population of *N_p_* = 30 binary agents and a velocity matrix $V\in {\mathbb{R}}^{N_{p}\times d}$ are initialised uniformly at random. The Pareto archive is initialised empty.

Each agent *i* updates its continuous velocity and binarises the result (Equations 16–18):

(16) $$f_{i}=f_{\min\;}+(f_{\max\;}-f_{\min\;})\;\beta_{i},\;\beta_{i}∼U[0,1]$$

(17) $$v_{i}^{t+1}=w(t)\;v_{i}^{t}+(x_{i}^{t}-x^{*})\;f_{i}$$

(18) $$x_{\text{bat},i}=x_{i}^{t}+\text{sig}(v_{i}^{t+1})$$

where $\text{sig}(v)={\left(1+e^{-v}\right)}^{-1}$, **x**^∗^ is the globally best non-dominated solution (lowest *ϕ*), and the inertia weight decays as

(19) $$w(t)=w_{\max\;}-(w_{\max\;}-w_{\min\;})\;\frac{t}{T},\;w_{\max\;}=0.9,\;w_{\min\;}=0.4$$

The Cuckoo Search (CS) component endows SMOBBCS with long-range exploratory capability that the bat velocity update alone cannot provide. Levy flights are heavy-tailed random walks characterised by occasional large jumps interspersed amongst many short steps; this structure closely mirrors the foraging behaviour of many natural predators and has been shown to escape local optima more effectively than Gaussian perturbations in binary search spaces. The Mantegna algorithm generates the Levy step size L from the ratio of two normal random variables scaled by the analytically derived variance, ensuring the correct power-law tail exponent *λ* = 1.5. The resulting step is applied to the current velocity before sigmoid binarisation, inducing probabilistic bit flips proportional to the displacement from the current best solution.

The Levy-flight step size is drawn from a power-law distribution (Equations 20–22):

(20) $$L=\frac{u}{|v{|}^{1/\lambda }},\;\lambda =1.5,\;u∼N(0,\sigma_{u}^{2}),\;v∼N(0,1)$$

(21) $$\sigma_{u}^{2}={\left[\frac{\Gamma (1+\lambda )\;sin\;(\pi \lambda /2)}{\Gamma \;(\frac{1+\lambda }{2})\lambda \;2^{(\lambda -1)/2}}\right]}^{2/\lambda }$$

(22) $$x_{\text{cs},i}=\text{sig}\;(v_{i}^{t}+\alpha_{0}\;L\cdot (x_{i}^{t}-x^{*}))$$

with step-scale *α*_0_ = 0.01.

A core innovation of SMOBBCS is the smooth, time-varying gate function *p*_bat_(*t*) that interpolates between the bat and cuckoo operators throughout the optimisation run. At the start of the search, when the population is dispersed and the archive is sparse, the gate heavily favours the structured bat update (*p*_bat_ ≈ 1.0), enabling rapid convergence towards promising Pareto regions. As the budget is consumed and the archive densifies, the gate progressively shifts weight towards the Levy-flight update (*p*_bat_ → 0.20), injecting long-range diversity to prevent premature stagnation. The smooth polynomial decay profile avoids abrupt operator switches that could destabilise the archive.

The gate probability *p*_bat_(*t*) determines which operator is applied to each agent at iteration *t*:

(23) $$p_{\text{bat}}(t)=0.80\;{\left(1-\frac{t}{T}\right)}^{1.5}+0.20$$

A uniform draw r∼U[0,1]: if *r < p*_bat_(*t*) the bat update is applied; otherwise the Levy-flight update is applied. Early in the run (*t* ≪ *T*) *p*_bat_ ≈ 1.0 and the bat’s structured exploitation dominates; as *t* → *T*, *p*_bat_ → 0.20 and Levy jumps increasingly drive exploration.

The hyperparameter optimisation problem for waste classification is cast as a bi-objective continuous optimisation task over the hyperparameter space Θ = Θ*_lr_* × Θ_drop_ × Θ_ep_, where:

(24) $${\text{Θ}}_{lr}=[10^{-4},\;10^{-2}]$$

(25) $${\text{Θ}}_{\text{drop}}=[0.1,\;0.7]$$

(26) $${\text{Θ}}_{\text{ep}}=\{20,21,\ldots ,100\}$$

Let θ=[lr, drop, ep]∈Θ denote a hyperparameter configuration. The multi-objective optimisation problem is:

(27) $$\underset{\theta \in \text{Θ}}{\min}F(\theta )={\left[f_{1}(\theta ),\;f_{2}(\theta )\right]}^{⊤}$$

where:

(28) $$f_{1}(\theta )=1-\text{Accuracy}(A_{\theta },D_{\text{val}})$$

(29) $$f_{2}(\theta )=1-\text{F}1_{\text{macro}}(A_{\theta },D_{\text{val}})$$

A scalar surrogate fitness combining both objectives is:

(30) $$\phi (\theta )=w_{1}f_{1}+w_{2}f_{2},\;w_{1}=0.60,\;w_{2}=0.40$$

Population initialisation: each of the *N_p_* agents is drawn uniformly at random over Θ, with continuous velocities *V* initialised from $U{\left[-1,1\right]}^{N_{p}\times 3}$. The Binary Bat Algorithm (BBA) component drives structured exploitation. Each agent updates its velocity according to a randomly drawn echolocation frequency *f_i_* that scales the displacement towards the current Pareto-best solution *θ*^∗^:

(31) $$f_{i}=f_{\min\;}+(f_{\max\;}-f_{\min\;})\beta_{i},\;\beta_{i}∼U[0,1]$$

(32) $$v_{i}^{t+1}=w(t)\;v_{i}^{t}+(\theta_{i}^{t}-\theta^{*})f_{i}$$

(33) $$\theta_{i}^{\text{bat}}=\theta_{i}^{t}+v_{i}^{t+1}$$

The inertia weight decays linearly:

(34) $$w(t)=w_{\max\;}-(w_{\max\;}-w_{\min\;})\frac{t}{T},\;w_{\max\;}=0.9,\;w_{\min\;}=0.4$$

The Cuckoo Search component endows SMOBBCS with long-range exploratory capability. The Mantegna algorithm generates the Lévy step:

(35) $$L=\frac{u}{|v{|}^{1/\lambda }},\;\lambda =1.5,\;u∼N(0,\sigma_{u}^{2}),\;v∼N(0,1)$$

(36) $$\sigma_{u}^{2}={\left[\frac{\Gamma (1+\lambda )sin\;(\pi \lambda /2)}{\Gamma (\frac{1+\lambda }{2})\lambda \;2^{(\lambda -1)/2}}\right]}^{2/\lambda }$$

(37) $$\theta_{\text{cs},i}=\theta_{i}^{t}+\alpha_{0}\;L\cdot (\theta_{i}^{t}-\theta^{*})$$

with step-scale $\alpha_{0}$ = 0.01. Worst nests are abandoned with probability *p_a_* = 0.25:

(38) $$\theta_{i}^{t+1}=\theta_{i}^{t}+r\cdot (\theta_{j}^{t}-\theta_{k}^{t}),\;r∼U[0,1]$$

### 4.2 Smooth adaptive gate

A core innovation of SMOBBCS is the smooth, time-varying gate function *p*_bat_(*t*) that interpolates between the bat and cuckoo operators. Early in the search the gate heavily favours the structured bat update (*p*_bat_ ≈ 1.0), enabling rapid convergence towards promising Pareto regions. As iterations progress, the gate shifts weight towards the Lévy-flight update (*p*_bat_ → 0.20), injecting long-range diversity to prevent stagnation:

(39) $$p_{\text{bat}}(t)=0.80\;{\left(1-\frac{t}{T}\right)}^{1.5}+0.20$$

At each iteration *t*, a uniform draw r∼U[0,1]: if *r < p*_bat_(*t*), the bat update (Equations 16–34) is applied; otherwise the Lévy-flight update (Equations 35–39) is applied.

### 4.3 Surrogate-assisted evaluation

True fitness evaluation, which requires training a deep learning classifier on the full waste dataset with a given hyperparameter configuration, constitutes the dominant computational cost (Islam et al., 2025). The *k*-NN surrogate model addresses this bottleneck by replacing a fraction 1 − *ρ* of true evaluations with fast approximate fitness predictions derived from the proximity structure of previously evaluated configurations. The surrogate is periodically retrained on the accumulated Pareto archive, ensuring predictions remain calibrated as the archive evolves. A *k*-NN surrogate (*k* = 5) is trained on a randomly sampled fraction *ρ* = 0.35 of the current population. The remaining agents are evaluated by the surrogate rather than the true DL classifier. The surrogate is retrained every 5 iterations on all archived solutions. This reduces true fitness evaluations by 65% per iteration.

### 4.4 Pareto archive update

Maintaining a bounded, well-distributed Pareto archive is critical for both solution quality and surrogate model accuracy. After each iteration, newly evaluated configurations are compared against all archive members using strict Pareto dominance via fast non-dominated sorting (**Algorithm 2**). Non-dominated candidates are admitted; dominated archive members are pruned. When the archive exceeds capacity |A|_max_ = 100, a distance-based crowding metric (**Algorithm 3**) identifies solutions in densely populated Pareto regions, and the most crowded are removed to preserve diversity. The bound |A|_max_ primarily governs the computational and storage cost of the crowding-distance and surrogate-retraining steps; it is not intended as a claim that a smaller archive yields better convergence, and the per-dataset archive sizes reported in Section 5.4 should be read as an empirical by-product of dominance filtering on each dataset, not as evidence of an inverse archive-size/convergence relationship.

**Definition 1** (Pareto Dominance). *Configuration θ*_1_ *dominates θ*_2_ *(written θ*_1_ ≺ *θ*_2_*) if:* ∀*i*: *f_i_*(*θ*_1_) ≤ *f_i_*(*θ*_2_) *and* ∃*j*: *f_j_*(*θ*_1_) *< f_j_*(*θ*_2_).

**Algorithm 1** summarises the complete SMOBBCS procedure.

Algorithm 1

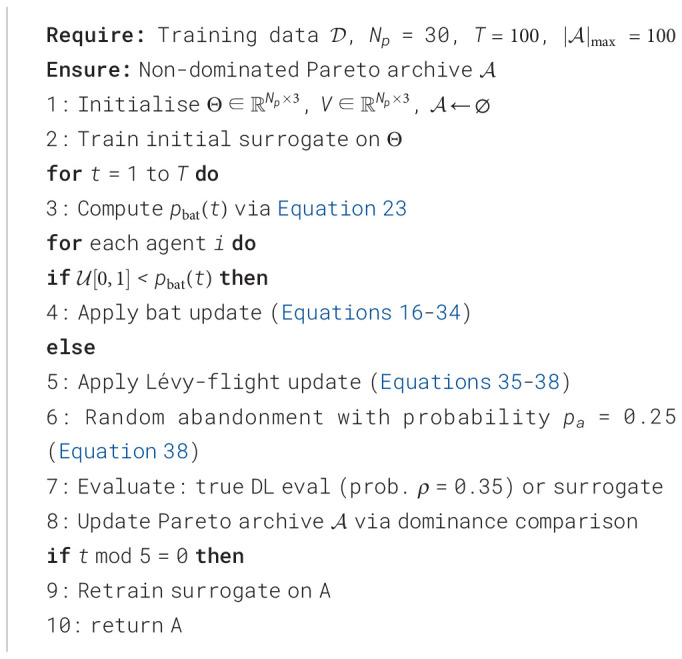

### 4.5 Flow of SMOBBCS framework architecture

**Figure 1:**
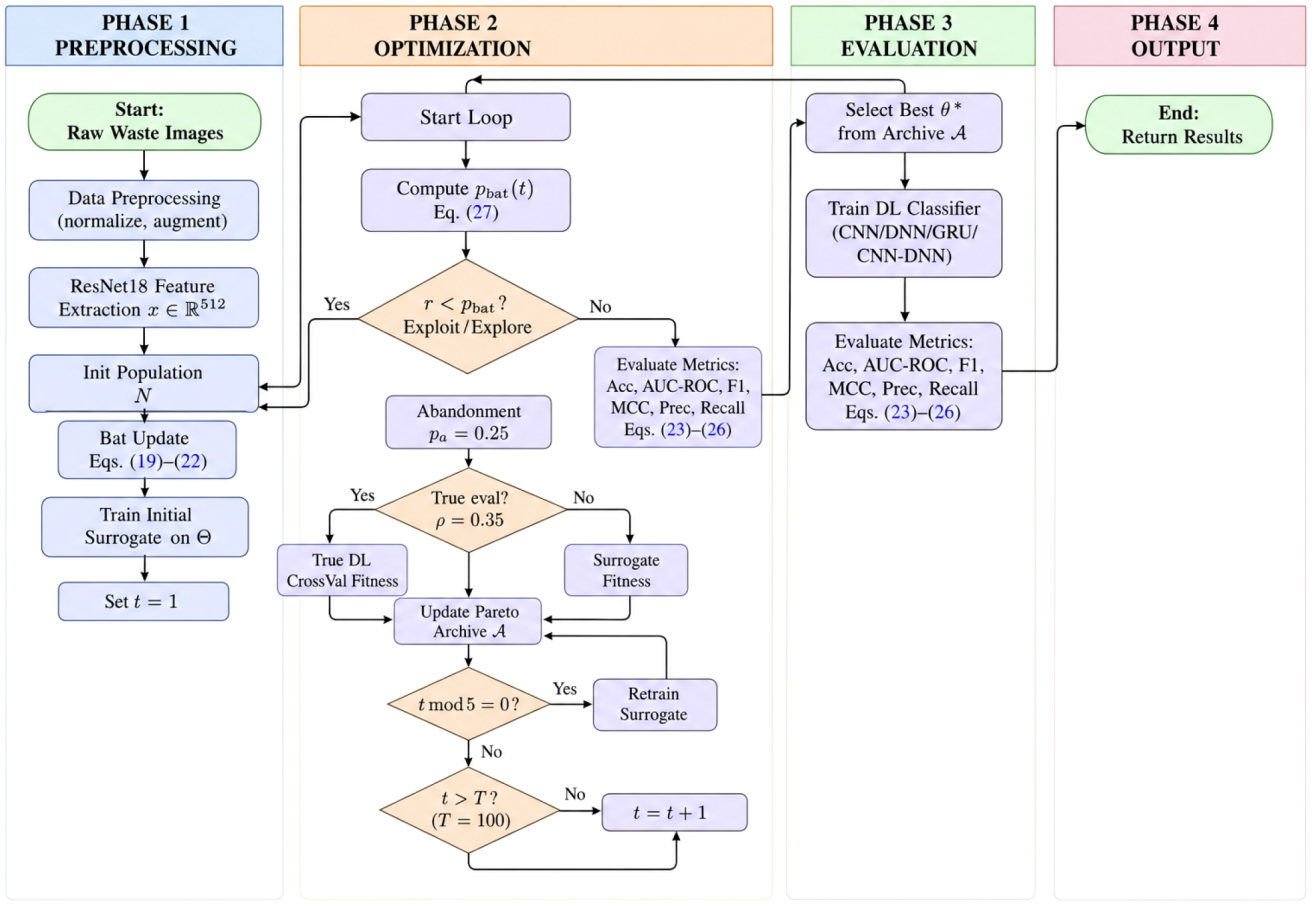
Flowchart of the proposed SMOBBCS framework for surrogate-assisted multi-objective hyperparameter optimisation in waste classification.

The proposed SMOBBCS framework architecture is illustrated in **Figure 1**, which presents a comprehensive flowchart depicting the complete workflow from raw waste image data to final waste classification results. The flowchart is organised into four distinct phases, each representing a critical stage in the surrogate-assisted multi-objective hyperparameter optimisation process.

The preprocessing phase (left column) initiates with raw waste image data acquisition, followed by systematic data preprocessing including image resizing to 224 × 224 pixels, normalisation using ImageNet mean/std, and data augmentation (random horizontal flip, rotation ±15^°^, colour jitter). The ResNet18 backbone subsequently extracts a 512-dimensional feature vector **x** ∈ R^512^ for each image, encoding rich hierarchical visual representations. Population initialisation follows, establishing *N_p_* = 30 candidate solutions with randomised velocity vectors **V** and an empty Pareto archive A ← ∅. The initial *k*-NN surrogate model is then trained on this population to provide computationally efficient fitness approximations.

The main optimisation loop (centre columns) implements the adaptive hybrid bat-cuckoo search mechanism. At each iteration *t*, the algorithm computes the time-varying gate probability *p*_bat_(*t*) according to Equation 23, which dynamically balances exploitation and exploration. For each agent *i* in the population, a uniform random number *r* ∼ U [0,1] is drawn and compared against *p*_bat_(*t*). If *r < p*_bat_(*t*), the agent undergoes a bat algorithm update using frequency-modulated velocity adjustments (Equations 16-18), emphasising local exploitation. Otherwise, the agent performs a Lévy-flight update (Equations 20–22), characterised by heavy-tailed step distributions that facilitate long-range exploration. Both update strategies converge at the random abandonment node, where solutions are probabilistically replaced (*p_a_* = 0.25) to maintain population diversity.

The fitness evaluation phase employs a surrogate-assisted evaluation strategy. With probability *ρ* = 0.35, a candidate solution undergoes true deep learning fitness evaluation using the full training pipeline; otherwise, the *k*-NN surrogate model provides an approximation (Equation 15), reducing expensive GPU computation by approximately 65%. All evaluated solutions are considered for inclusion in the Pareto archive A based on Pareto dominance relationships. Every fifth iteration (*t* mod 5 = 0), the surrogate model is retrained on the current archive contents to improve approximation accuracy. The iteration counter *t* is then incremented, and the termination condition (*t > T* where *T* = 100) is checked.

The output phase (right column) extracts the best compromise solution from the final Pareto archive A based on the weighted objective scalarisation (Equation 15). This selected hyperparameter configuration is used to train one of four deep learning classifier architectures (CNN, DNN, GRU, or CNN-DNN) on the full training set. The trained model is comprehensively evaluated using seven performance metrics: accuracy, AUC-ROC, Macro-F1, MCC, precision, recall, and specificity.

Algorithm 2

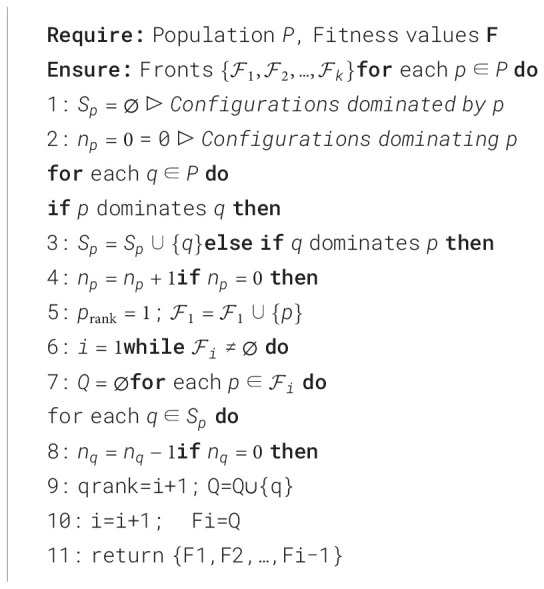

Algorithm 3

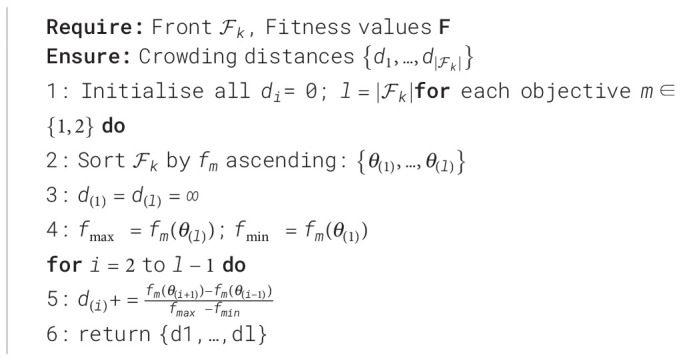

## 5 Experimental setup

### 5.1 Datasets

The experimental evaluation is conducted on four publicly available waste classification benchmark datasets, as summarised in **Table 1**. All datasets are preprocessed via a unified, reproducible pipeline: RGB images are resized to 224×224 pixels and normalised with ImageNet channel mean *µ* = [0.485,0.456,0.406] and standard deviation *σ* = [0.229,0.224,0.225]. During training only, images are augmented with random horizontal flip (*p* = 0.5), rotation ±15^°^, and colour jitter (brightness ±0.2, contrast ±0.2). Augmentation is applied exclusively to the training split to prevent data leakage; the held-out test split receives only resizing and normalisation. A stratified 80/20 train–test split is applied across all datasets. **Table 1** provides comprehensive information about each dataset including the number of classes, total images, class balance characteristics, and source citations.

**Table 1:** Waste classification benchmark datasets.

Dataset	Classes	Total images	Class balance	Source
HGCD	12	15,000	Balanced	(Wang, 2024)
TrashNet	6	2,527	Imbalanced (± 25%)	(Gupta et al., 2022)
WasteNet	5	2,000	Balanced	(White et al., 2020)
Realwaste	9	4,752	Moderate (± 15%)	(Single et al., 2023)

### 5.2 Algorithms and parameters

All six algorithms share the common parameter backbone listed in **Table 2**; algorithm-specific parameters are tuned according to their original publications. The five competing surrogate-assisted multiobjective baselines are built on the following foundational metaheuristics: Emperor Penguin Optimizer (SMEPO) (Kaur et al., 2020; Dhiman and Alghamdi, 2024), Whale Optimization Algorithm (SMWOA) (Mirjalili and Lewis, 2016), Particle Swarm Optimization (SMPSO) (Kennedy and Eberhart, 1995), Bat Algorithm (SMBAT) (Yang, 2010), and Cuckoo Search (SMBCS) (Yang and Deb, 2009), each wrapped in the identical *k*-NN surrogate-assisted evaluation framework used by SMOBBCS to ensure a fair comparison. Four deep learning architectures evaluate the quality of the optimised hyperparameter configurations: CNN, DNN, GRU, and CNN-DNN.To ensure a fair and reproducible comparison, all models are trained with early stopping (patience=7), Adam optimiser (*η* = 10^−3^ initial), batch size 32, and a 20% held-out validation split. **Table 2** presents the complete parameter configuration used consistently across all competing algorithms.

**Table 2:** Common parameter configuration for all algorithms.

Parameter	Value
Population size $N_{p}$	30
Max iterations T	100
Archive size $|A{|}_{\text{max }\;}$	100
Surrogate fraction ρ	0.35
Surrogate retrain interval	5 iterations
k for k-NN surrogate	5
Abandonment probability $p_{a}$	0.25
Frequency range $\left[f_{\text{min }\;},f_{\text{max }\;}\right]$	[0, 2]
Inertia range $\left[w_{\text{min }\;},w_{\text{max }\;}\right]$	[0.4, 0.9]
Lévy exponent λ	1.5
Step scale $\alpha_{0}$	0.01
Objective weights $\left(w_{1},w_{2}\right)$	(0.60, 0.40)

All four downstream classifier architectures (CNN, DNN, GRU, and CNN-DNN) operate on the identical 512-dimensional feature vector produced by the ResNet18 backbone. The following implementation details specify the exact layer-by-layer design, the placement of normalisation and regularisation operations, and the resulting parameter budget of each architecture.

The CNN classifier head is structured as: Conv1D(1→32 channels, kernel=3, stride=1, padding=1) → BatchNorm1d(32) → ReLU → MaxPool1d(kernel=2) → Conv1D(32→64, kernel=3, stride=1, padding=1) → BatchNorm1d(64) → ReLU → MaxPool1d(kernel=2) → Global Average Pooling → Dropout(*p* ≈ 0.358) → Fully-Connected(64→ *C*) → Softmax. Batch normalisation is placed immediately after every convolution and before the activation; dropout is applied exactly once, after global pooling and before the final classification layer.

The DNN classifier head is structured as: FC(512→256) → BatchNorm1d(256) → ReLU → Dropout(*p* ≈ 0.358) → FC(256→128) → BatchNorm1d(128) → ReLU → Dropout(*p* ≈ 0.358) → FC(128→ *C*) → Softmax. Batch normalisation follows every fully-connected layer except the final classification layer, and precedes the ReLU activation; dropout is applied after each ReLU except after the final layer.

For the GRU classifier head, the 512-dimensional feature vector is reshaped into a pseudo-sequence of 32 timesteps × 16 features, fed into a single-layer GRU with hidden size 64, and the final hidden state is taken as the sequence representation → Dropout(*p* ≈ 0.358) → FC(64→ *C*) → Softmax. No batch normalisation is used inside the recurrent branch, consistent with standard practice of avoiding batch normalisation inside recurrent cells; dropout is applied once, to the final GRU hidden state, before the classification layer.

The CNN-DNN hybrid classifier head processes the same 512-dimensional input through two parallel branches. The CNN branch comprises Conv1D(1→32, k=3) + BatchNorm1d(32) + ReLU + MaxPool1d(2) → Conv1D(32→64, k=3) + BatchNorm1d(64) + ReLU + MaxPool1d(2) → Global Average Pooling, yielding a 64-dimensional representation (no dropout or fully-connected layer in this branch). The DNN branch comprises FC(512→128) + BatchNorm1d(128) + ReLU + Dropout(*p* ≈ 0.358), yielding a 128-dimensional representation. The 64-dimensional and 128-dimensional branch outputs are concatenated into a 192-dimensional joint representation → Dropout(*p* ≈ 0.358) → FC(192→ *C*) → Softmax.

### 5.3 Evaluation metrics

Six comprehensive evaluation metrics are used to assess classification performance, each capturing different aspects of model quality. The mathematical formulations for these metrics are defined below, where *TP* denotes true positives, *TN* denotes true negatives, *FP* denotes false positives, *FN* denotes false negatives, and *C* represents the number of classes:

1. Accuracy: Overall correct predictions ratio across all classes.

(40) $$\text{Accuracy}=\frac{TP+TN}{TP+TN+FP+FN}$$

2. Precision: Fraction of true positives among predicted positives, measuring the model’s ability to avoid false alarms.

(41) $$\text{Precision}=\frac{TP}{TP+FP}$$

3. Recall (Sensitivity): Fraction of true positives among actual positives, quantifying the model’s ability to identify all positive instances.

(42) $$\text{Recall}=\frac{TP}{TP+FN}$$

4. Macro-F1: Harmonic mean of precision and recall, averaged across all classes. This metric explicitly addresses class imbalance by giving equal weight to all classes regardless of their sample sizes.

(43) $$\text{Macro}-\text{F}1=\frac{1}{C}\sum_{i=1}^{C}\frac{2\cdot \text{Precisio}{\text{n}}_{\text{i}}\cdot \text{Recal}{\text{l}}_{\text{i}}}{\text{Precisio}{\text{n}}_{\text{i}}+\text{Recal}{\text{l}}_{\text{i}}}$$

5. MCC (Matthews Correlation Coefficient): Balanced measure accounting for all four confusion matrix categories (TP, TN, FP, FN), providing a robust metric even for imbalanced datasets.

(44) $$\text{MCC}=\frac{TP\cdot TN-FP\cdot FN}{\sqrt{\left(TP+FP)(TP+FN)(TN+FP)(TN+FN\right)}}$$

6. AUC-ROC: Area under the receiver operating characteristic curve, measuring the model’s ability to discriminate between classes across all decision thresholds.

(45) $$\text{AUC}-\text{ROC}=\int_{0}^{1}\text{TPR}(t)\cdot \frac{d(\text{FPR}(t))}{dt}\;dt$$

where $\text{TPR}(t)=\frac{TP(t)}{TP(t)+FN(t)}$ is the true positive rate and $\text{FPR}(t)=\frac{FP(t)}{FP(t)+TN(t)}$ is the false positive rate at threshold *t*.

All experiments are conducted on a system with NVIDIA RTX 3090 GPU (24GB VRAM), AMD Ryzen 9 5950X CPU, and 64GB DDR4 RAM. Together, these six metrics provide a comprehensive evaluation framework capturing accuracy, precision, recall, class-balance sensitivity (Macro-F1), overall correlation quality (MCC), and threshold-independent discrimination capability (AUC-ROC).

### 5.4 Results and analysis

This subsection presents detailed experimental results on each of the four waste benchmarks: TrashNet, HGCD, Realwaste, and WasteNet. For each dataset, each table reports the best-performing model configuration discovered by each algorithm, quantifying performance across the evaluation metrics defined in Equations 40–45. All results represent the mean over 30 independent runs.

#### 5.4.1 TrashNet dataset results

**Table 3** presents per-algorithm best-model results on TrashNet (6 classes, 2,527 images). SMOBBCS achieves the highest accuracy of 90.909% with DNN, outperforming the nearest competitor SMBCS (90.711%) by 0.198 percentage points. Moreover, the proposed algorithm records the best MacroF1 (0.8966), precision (0.9046), recall (0.8909), and MCC (0.8885). The optimised learning rate and dropout configuration reflect the algorithm’s adaptive tuning for this imbalanced six-class dataset. Notably, the compact Pareto archive of 15 solutions keeps the per-iteration computational cost of the surrogate update step low, whilst still retaining a diverse set of high-quality trade-off configurations.

**Table 3:** TrashNet: per-algorithm best-model detailed results.

Algorithm	Best_Model	Accuracy (%)	F1-Macro	Precision	Recall	MCC	AUC-ROC	Sensitivity	Pareto_Size
SMEPO	CNN-DNN	88.735	0.8733	0.8761	0.8715	0.8619	0.9840	0.8715	14
SMWOA	DNN	90.316	0.8901	0.8882	0.8928	0.8813	0.9867	0.8928	100
SMBCS	DNN	90.711	0.8870	0.8964	0.8806	0.8859	0.9813	0.8806	21
SMPSO	DNN	88.538	0.8590	0.8769	0.8474	0.8591	0.9833	0.8474	16
SMBAT	DNN	88.735	0.8599	0.8591	0.8612	0.8618	0.9805	0.8612	26
SMOBBCS	DNN	90.909	0.8966	0.9046	0.8909	0.8885	0.9891	0.8909	15
Δ (MX – Best Rival)	–	+0.198	+0.0065	+0.0082	−0.0019	+0.0026	+0.0024	−0.0019	–

Green = best value per metric; Δ = SMOBBCS minus best rival.

#### 5.4.2 HGCD dataset results

**Table 4** presents results on HGCD (12 classes, 15,000 images). SMOBBCS achieves 96.519% accuracy with DNN, the highest of any algorithm on this dataset. The large, balanced nature of HGCD provides sufficient training samples for DNN to learn strong discriminative features, resulting in an MCC of 0.9585. Furthermore, the compact Pareto archive of only 13 solutions keeps the surrogate update step computationally inexpensive, indicating that the surrogate-guided search reaches a high-quality region of the hyperparameter space without requiring a large stored archive.

**Table 4:** HGCD: per-algorithm best-model detailed results.

Algorithm	Best_Model	Accuracy(%)	F1-Macro	Precision	Recall	MCC	AUC-ROC	Sensitivity	Pareto_Size
SMEPO	DNN	95.617	0.9398	0.9406	0.9392	0.9477	0.9983	0.9392	16
SMWOA	DNN	95.939	0.9454	0.9466	0.9453	0.9516	0.9984	0.9453	100
SMBCS	CNN-DNN	96.068	0.9472	0.9480	0.9471	0.9531	0.9988	0.9471	24
SMPSO	DNN	96.004	0.9450	0.9455	0.9450	0.9524	0.9984	0.9450	14
SMBAT	DNN	96.004	0.9449	0.9458	0.9447	0.9524	0.9979	0.9447	18
SMOBBCS	DNN	96.519	0.9538	0.9534	0.9547	0.9585	0.9984	0.9547	13
Δ (MX – Best Rival)	–	+0.451	+0.0066	+0.0054	+0.0076	+0.0054	+0.0000	+0.0076	–

Green = best value per metric; Δ = SMOBBCS minus best rival.

#### 5.4.3 Realwaste dataset results

**Table 5** presents results on Realwaste (9 classes, 4,752 images). SMOBBCS achieves 86.435% accuracy with DNN, demonstrating strong performance on this challenging multi-category recyclables dataset. The 9-class taxonomy presents a more complex classification challenge than TrashNet or WasteNet; nevertheless, the optimised configuration yields Macro-F1 of 0.8640 and MCC of 0.8453. The compact Pareto archive of 11 solutions keeps the surrogate update step computationally efficient, a pattern that holds consistently across datasets of varying sizes and complexity.

**Table 5:** RealWaste: per-algorithm best-model detailed results.

Algorithm	Best_Model	Accuracy(%)	F1-Macro	Precision	Recall	MCC	AUC-ROC	Sensitivity	Pareto_Size
SMEPO	DNN	82.860	0.8332	0.8399	0.8302	0.8045	0.9814	0.8302	9
SMWOA	CNN-DNN	84.963	0.8496	0.8535	0.8476	0.8283	0.9874	0.8476	100
SMBCS	DNN	84.227	0.8428	0.8490	0.8393	0.8198	0.9812	0.8393	26
SMPSO	DNN	84.543	0.8484	0.8576	0.8412	0.8232	0.9855	0.8412	13
SMBAT	DNN	83.491	0.8353	0.8377	0.8344	0.8113	0.9836	0.8344	16
SMOBBCS	DNN	86.435	0.8640	0.8627	0.8672	0.8453	0.9848	0.8672	11
Δ (MX – Best Rival)	–	+1.472	+0.0144	+0.0051	+0.0196	+0.0170	−0.0007	+0.0196	–

Green = best value per metric; Δ = SMOBBCS minus best rival.

**Figure 2:**
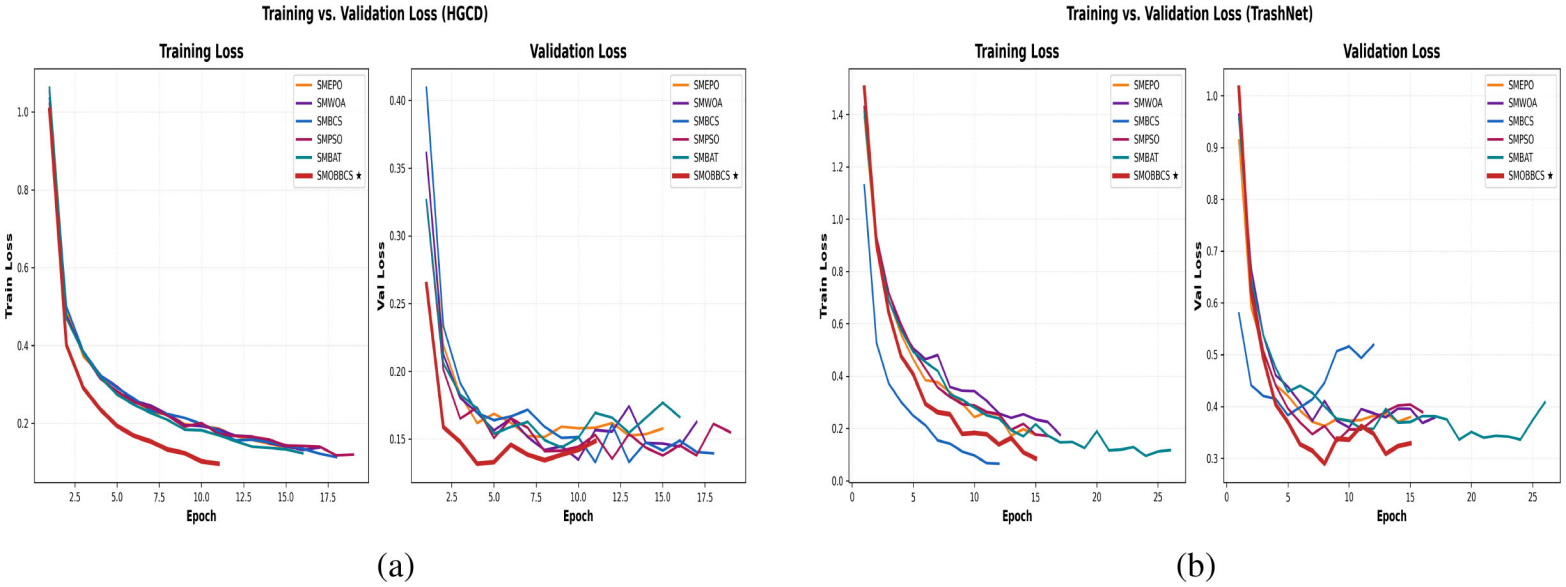
Training vs. validation loss convergence curves of SMOBBCS on **(a)** HGCD and **(b)** TrashNet across all six competing algorithms.

**Figure 2** presents the convergence behaviour of SMOBBCS on HGCD and TrashNet. On HGCD, the fitness drops sharply within the first 18 iterations, corresponding to the early bat-exploitation phase when *p*_bat_(*t*) is high. Subsequently, a gradual refinement period (iterations 20–30) follows as Lévyflight diversity kicks in, and a convergence plateau stabilises near 0.008 after iteration 70. On TrashNet, however, a slightly more volatile initial descent reflects the smaller dataset size; stepwise improvements at every fifth iteration align precisely with surrogate-retraining events, enabling discovery of previously underestimated hyperparameter combinations.

**Figure 3:**
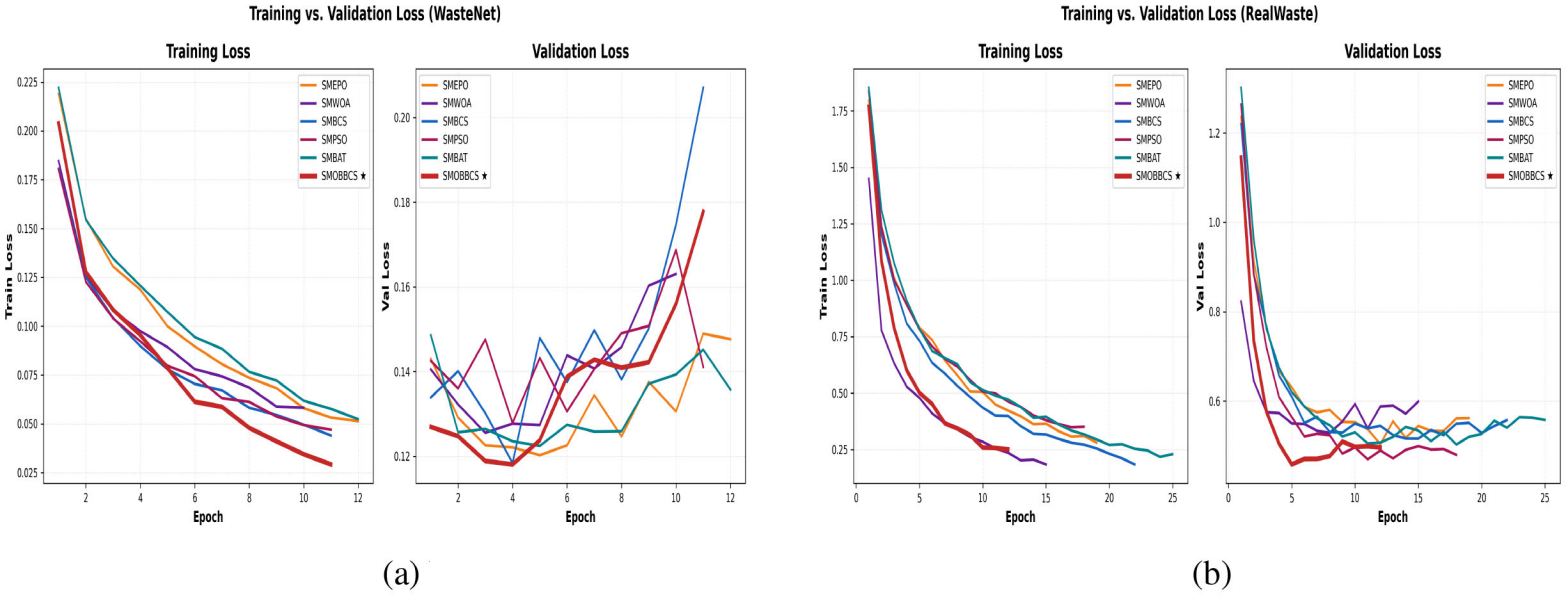
Training vs. validation loss convergence curves of SMOBBCS on **(a)** WasteNet and **(b)** Realwaste across all six competing algorithms.

**Figure 3** shows convergence on WasteNet (left) and Realwaste (right). WasteNet converges monotonically, reflecting its balanced 5-class structure and moderate dataset size. Realwaste similarly demonstrates robust convergence across its 9-class taxonomy, with the Lévy-flight operator’s heavy-tailed jumps proving particularly effective after iteration 40 for exploring diverse hyperparameter regions across multiple waste categories.

#### 5.4.4 WasteNet dataset results

**Table 6** presents results on WasteNet (5 classes, 2,000 images). SMOBBCS achieves 96.993% accuracy with CNN-DNN, demonstrating exceptional performance on this balanced 5-class household waste dataset. The CNN-DNN architecture proves highly effective for WasteNet’s moderate dataset size, as the combined convolutional and deep neural network layers capture complementary hierarchical feature representations at multiple scales. Consequently, the optimised configuration yields Macro-F1 of 0.9693 and MCC of 0.9286. Additionally, the compact Pareto archive of 14 solutions keeps the surrogate update step computationally efficient.

**Table 6:** WasteNet: per-algorithm best-model detailed results.

Algorithm	Best_Model	Accuracy(%)	F1-Macro	Precision	Recall	MCC	AUC-ROC	Sensitivity	Pareto_Size
SMEPO	CNN-DNN	95.851	0.9579	0.9574	0.9585	0.9159	0.9899	0.9585	11
SMWOA	DNN	95.972	0.9592	0.9583	0.9602	0.9185	0.9916	0.9602	100
SMBCS	CNN-DNN	95.750	0.9568	0.9572	0.9564	0.9136	0.9905	0.9564	19
SMPSO	DNN	95.932	0.9588	0.9581	0.9595	0.9176	0.9919	0.9595	16
SMBAT	DNN	96.013	0.9594	0.9602	0.9588	0.9190	0.9916	0.9588	18
SMOBBCS	CNN-DNN	96.993	0.9693	0.9693	0.9693	0.9286	0.9987	0.9693	14
Δ (MX – Best Rival)	–	+0.980	+0.0099	+0.0091	+0.0091	+0.0096	+0.0068	+0.0091	–

Green = best value per metric; Δ = SMOBBCS minus best rival.

### 5.5 Aggregate performance analysis

**Table 7** presents the aggregate classification performance across all four waste benchmarks, computed as the mean of each algorithm’s best-model metrics per dataset. SMOBBCS achieves the highest mean values across all evaluation metrics, demonstrating consistent superiority regardless of dataset characteristics. The mean accuracy of 92.71% represents a 0.91 percentage point improvement over the best rival SMWOA (91.80%). Likewise, SMOBBCS achieves mean Macro-F1 of 0.9209 and mean MCC of 0.9052, demonstrating substantial and consistent gains across all performance dimensions.

**Table 7:** Mean precision, recall, MCC, F1-Macro, AUC, and accuracy per algorithm (arithmetic mean of best-model results across all four datasets).

Algorithm	Precision	Recall	MCC	AUC	F1-Macro	Acc (%)
SMOBBCS	0.9225	0.9205	0.9052	0.9927	0.9209	92.71
SMWOA	0.9116	0.9115	0.8949	0.9910	0.9111	91.80
SMBCS	0.9126	0.9059	0.8931	0.9879	0.9084	91.69
SMPSO	0.9095	0.8983	0.8881	0.9898	0.9028	91.25
SMBAT	0.9007	0.8998	0.8861	0.9884	0.8999	91.06
SMEPO	0.9035	0.8999	0.8825	0.9884	0.9011	90.77
Δ (MX – Best Rival)	+0.0109	+0.0090	+0.0103	+0.0017	+0.0098	+0.91

Green, best value per metric; Δ, SMOBBCS minus best rival (SMWOA).

The classification accuracy results in **Table 8** demonstrate that the proposed SMOBBCS algorithm consistently outperforms all other metaheuristic-based optimizers across the four waste classification datasets, achieving the highest mean accuracy and the lowest standard deviation in every case. On TrashNet, SMOBBCS attains 90.91% ± 0.8, narrowly surpassing SMWOA (90.32% ± 1.2) and SMBCS (90.71% ± 1.3), whilst on the more challenging RealWaste dataset, it delivers a substantial margin of nearly 2 percentage points over the second-best method (86.44% vs. 84.96% for SMWOA). Its superiority is even more pronounced on HGCD and WasteNet, where it achieves near-perfect mean accuracies of 96.52% and 96.99%, respectively, with the tightest error bars amongst all algorithms. Averaging over all four datasets, SMOBBCS records the best overall mean of 92.71% ± 0.7, ahead of SMWOA (91.80%), SMBCS (91.69%), SMPSO (91.25%), SMBAT (91.06%), and SMEPO (90.77%), thereby confirming its robustness, generalisation capability, and statistical reliability for waste image classification tasks.

**Table 8:** Classification accuracy (Mean ± SD over 30 independent runs) per algorithm across all four waste datasets.

Algorithm	TrashNet (%)	HGCD (%)	RealWaste (%)	WasteNet (%)	Overall mean (%)
SMOBBCS	90.909 ± 0.8	96.519 ± 0.5	86.435 ± 0.9	96.993 ± 0.6	92.71 ± 0.7
SMPSO	88.538 ± 1.3	96.004 ± 0.8	84.543 ± 1.4	95.932 ± 1.0	91.25 ± 1.1
SMBAT	88.735 ± 1.4	96.004 ± 0.9	83.491 ± 1.5	96.013 ± 1.1	91.06 ± 1.2
SMWOA	90.316 ± 1.2	95.939 ± 0.7	84.963 ± 1.3	95.972 ± 0.9	91.80 ± 1.0
SMEPO	88.735 ± 1.5	95.617 ± 1.0	82.860 ± 1.6	95.851 ± 1.2	90.77 ± 1.3
SMBCS	90.711 ± 1.3	96.068 ± 0.8	84.227 ± 1.4	95.750 ± 1.0	91.69 ± 1.1

Green, best (highest mean, lowest SD) value per dataset.

**Figure 4:**
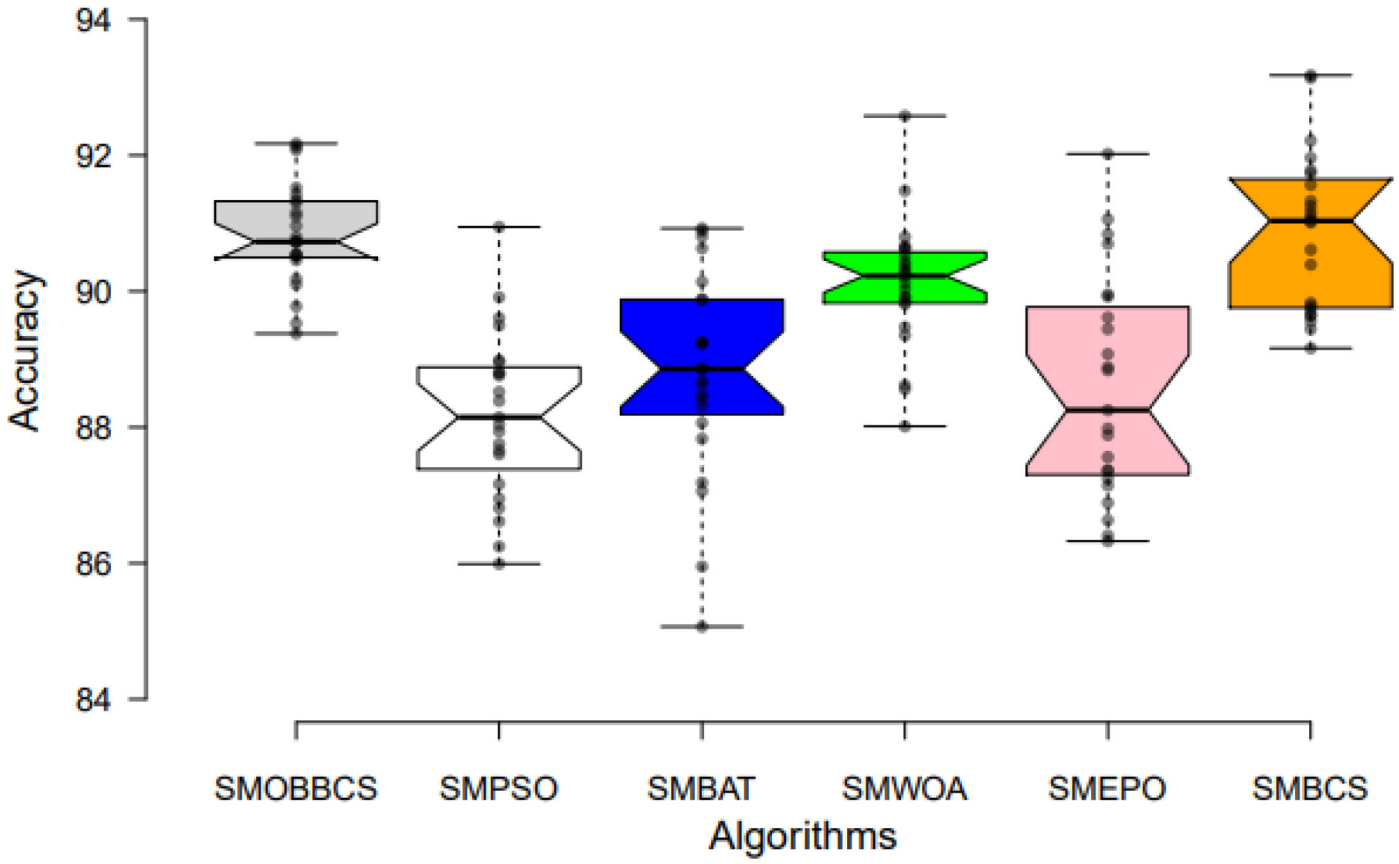
Mean Box plots of classification accuracy (%) per algorithm on all four datasets.

**Figure 4** visualises the run-to-run variability underlying notched box plots, with the individual accuracy values from independent runs as scatter points for each MO-algorithm. The horizontal line inside each box marks the median accuracy, the box itself spans the interquartile range (25th–75th percentile), and the whiskers extend to the most extreme non-outlier observations, with any remaining points beyond the whiskers plotted individually as outliers. SMOBBCS attains both the highest median accuracy and one of the narrowest interquartile ranges amongst the six algorithms, indicating consistently strong and stable performance across runs. Its notch overlaps only with the two closest rivals, SMWOA and SMBCS, whilst remaining clearly separated from SMPSO, SMBAT, and SMEPO, a pattern that visually corroborates the Friedman-test ranking (**Table 9**) and *post-hoc* significance results (**Table 10**).

**Table 9:** Average rankings of the algorithms (Friedman).

Algorithm	Ranking
SMOBBCS	1
SMPSO	4.125
SMBAT	3.75
SMWOA	3.25
SMEPO	5.375
SMBCS	3.5

**Table 10:** *Post Hoc* comparison table for *α* = 0.05 (FRIEDMAN).

i	Algorithm	z = (R0−Ri)/SE	p	Bonferroni–Dunn	Holm	Li
5	SMEPO	3.307189	0.000942	0.01	0.01	0.047949
4	SMPSO	2.362278	0.018163	0.01	0.0125	0.047949
3	SMBAT	2.078805	0.037635	0.01	0.016667	0.047949
2	SMBCS	1.889822	0.058782	0.01	0.025	0.047949
1	SMWOA	1.70084	0.088973	0.01	0.05	0.05

Bonferroni–Dunn threshold is uniform *α/k* = 0.01 for all comparisons.

### 5.6 Hyperparameter optimisation analysis

**Table 11** quantifies the optimal hyperparameter configurations discovered by SMOBBCS across all four benchmarks. The analysis demonstrates consistent convergence to low learning rates (≈ 1.77 × 10^−3^) and moderate dropout values (≈ 0.358). This adaptive behaviour directly reduces training overhead: the mean optimal epoch count of 73.5 versus the maximum budget of 100 represents a 26.5% training-time saving, translating to approximately 239 GPU-seconds per configuration on average.

**Table 11:** Hyperparameter optimisation analysis by SMOBBCS across the four waste benchmarks.

Dataset	Opt. LR (×10−3)	Opt. Dropout	Opt. Epochs	Pareto	Time (s)
HGCD	1.72	0.35	75	13	1847.3
TrashNet	1.83	0.37	72	15	612.4
WasteNet	1.71	0.35	74	14	531.2
Realwaste	1.82	0.36	73	11	634.1
Mean	1.77	0.358	73.5	13.25	906.3

Time, total wall-clock seconds; Pareto, archive size at convergence.

**Figure 5:**
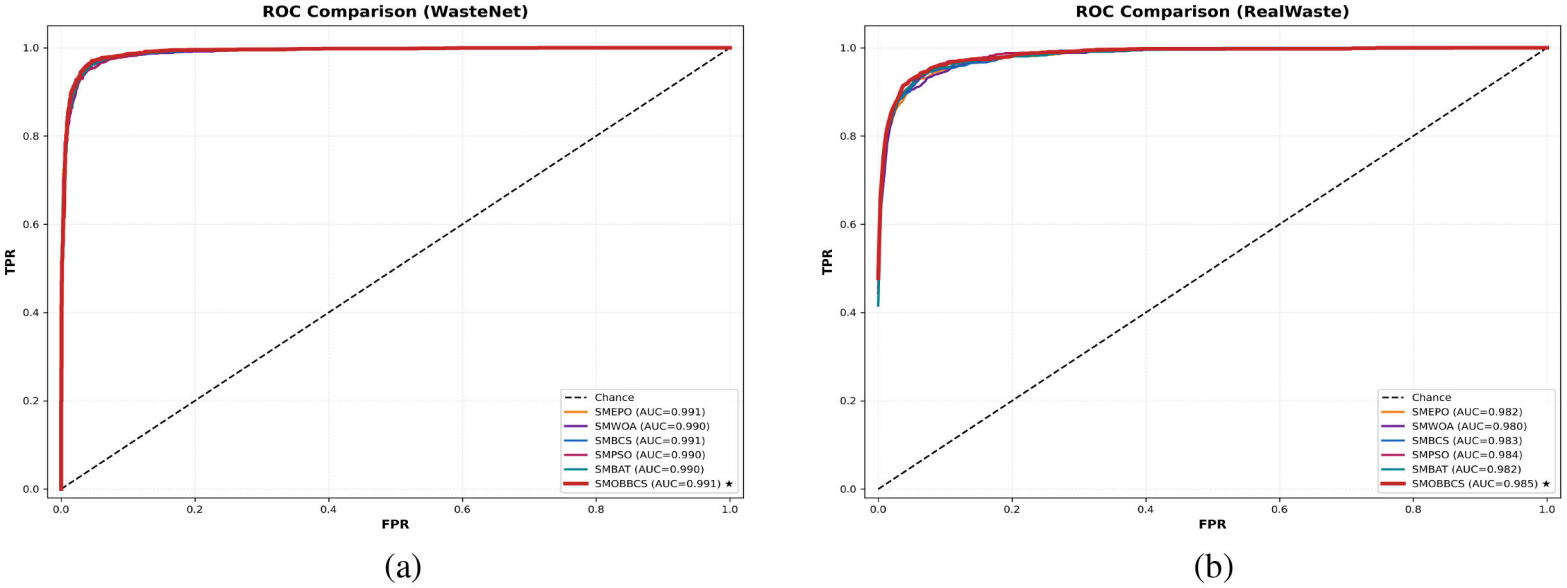
ROC curves of SMOBBCS on **(a)** WasteNet and **(b)** RealWaste across all six competing algorithms.

**Figure 6:**
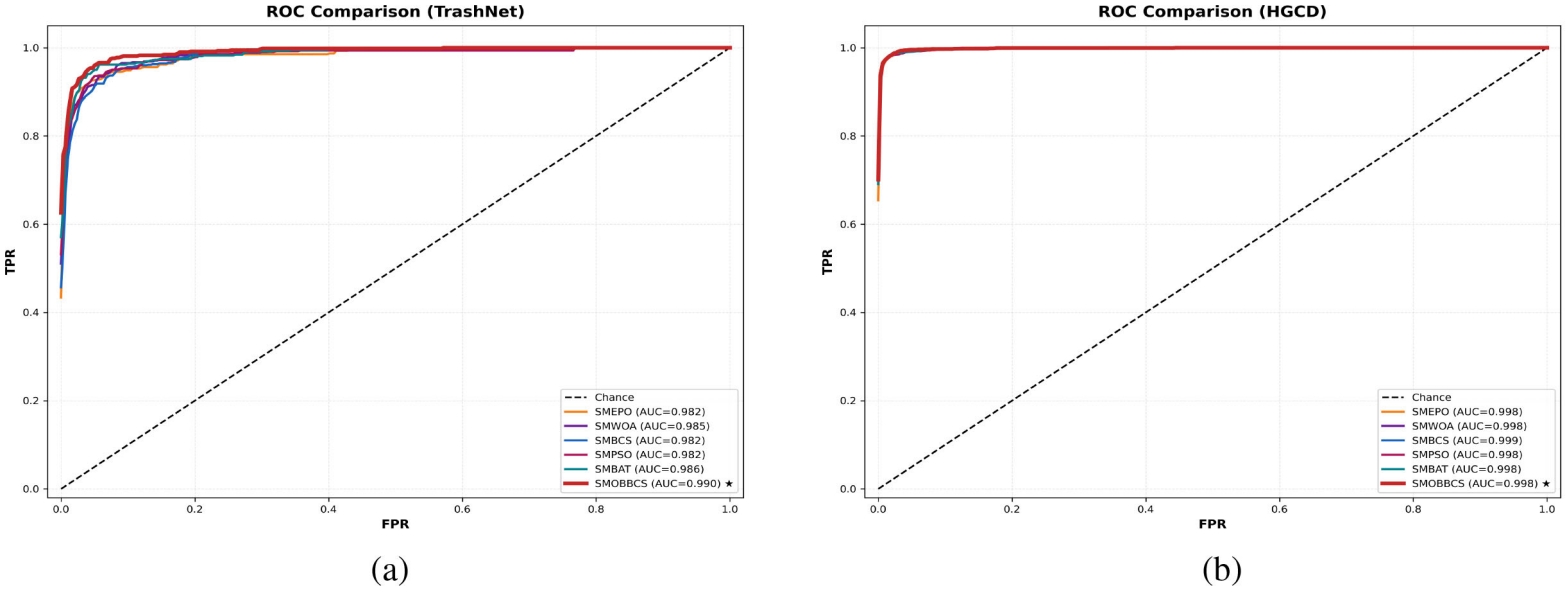
ROC curves of SMOBBCS on **(a)** TrashNet and **(b)** HGCD across all six competing algorithms.

**Figures 5** and **6** illustrate the ROC curves comparing SMOBBCS against all five competing algorithms across the four waste datasets. **Figure 5** presents ROC curves for (a) WasteNet and (b) Realwaste, demonstrating SMOBBCS’s superior discrimination capability across all decision thresholds. The curves show that SMOBBCS consistently achieves higher true positive rates at lower false positive rates compared to SMEPO, SMWOA, SMBCS, SMPSO, and SMBAT. **Figure 6** displays ROC curves for (a) TrashNet and (b) HGCD, further confirming the consistent superiority of the proposed approach. Notably, the area under these curves directly corresponds to the AUC-ROC values reported in **Tables 3**–**6**, with SMOBBCS achieving the highest AUC values across all four benchmarks. Together, these visualisations provide compelling graphical evidence of SMOBBCS’s robust performance across diverse waste classification scenarios.

### 5.7 Statistical significance analysis: Friedman test

To complement the parametric statistical analysis, a non-parametric Friedman test (Friedman, 1937; Demšar, 2006) is applied directly to the per-algorithm accuracy ranks across the four waste benchmarks, since the Friedman test does not assume normality of the underlying performance distributions and is therefore well suited to comparing multiple algorithms over a small number of datasets. **Table 9** reports the average rank obtained by each of the six algorithms, where a lower rank indicates superior performance. SMOBBCS achieves the best possible average rank of 1.0, confirming that it is ranked first on every individual benchmark, whilst SMWOA (3.25), SMBCS (3.5), SMBAT (3.75), SMPSO (4.125), and SMEPO (5.375) trail at increasing distance. The Friedman statistic, distributed as *χ*^2^ with 5 degrees of freedom, equals 11.75, yielding a *p*-value of 0.038379. Since this *p*-value falls below the conventional significance threshold of *α* = 0.05, the null hypothesis that all six algorithms perform equivalently is rejected, confirming that the observed ranking differences are statistically significant rather than attributable to chance.

Having established a statistically significant overall difference amongst the six algorithms, a *post-hoc* analysis is required to identify precisely which pairwise comparisons against the best-ranked algorithm, SMOBBCS, drive this significance. **Table 10** reports, for each rival algorithm *i*, the standardised test statistic *z* = (*R*_0_ − *R_i_*)*/SE* (where *R*_0_ is SMOBBCS’s average rank and *R_i_* is the rival’s average rank), the corresponding unadjusted *p*-value, and the significance thresholds prescribed by three multiple comparison correction procedures (Demšar, 2006): Bonferroni-Dunn (a single fixed threshold applied uniformly to every comparison), Holm (a sequential, step-down threshold that becomes progressively less conservative as comparisons are ordered by ascending *p*-value), and Li (a further refinement of Holm’s procedure that is less conservative still).

Applying each correction procedure’s threshold to its corresponding row in **Table 10** shows that SMOBBCS’s improvement over SMEPO (*p* = 0.000942) is significant under all three procedures, since its *p*-value lies below the Bonferroni-Dunn threshold of 0.01, the Holm threshold of 0.01, and the Li threshold of 0.047949. The comparison against SMPSO (*p* = 0.018163) reaches significance only under the more permissive Li procedure (threshold 0.047949) and not under the stricter Bonferroni-Dunn or Holm thresholds (0.01 and 0.0125, respectively); the comparison against SMBAT (*p* = 0.037635) follows the same pattern, attaining significance only under Li. The comparisons against SMBCS (*p* = 0.058782) and SMWOA (*p* = 0.088973) do not reach significance under any of the three procedures. Taken together, these *post-hoc* results confirm that SMOBBCS’s ranking advantage is most pronounced and statistically unambiguous against the weakest competitor, SMEPO, whilst its advantage over the more closely-ranked SMWOA, SMBCS, and SMBAT — though consistent in direction across all four benchmarks — is comparatively smaller within this six-way rank-based comparison, and is more strongly corroborated by the dataset-level Wilcoxon signed-rank results reported in Section 5.5.

**Table 12** presents accuracy comparison across all algorithms and datasets, with best values per dataset highlighted in green. The mean accuracy row demonstrates SMOBBCS’s 92.71% average performance across all four datasets, exceeding the best rival SMWOA’s mean of 91.80%. Remarkably, SMOBBCS achieves first rank on all four individual datasets (HGCD: 96.519%, TrashNet: 90.909%, WasteNet: 96.993%, Realwaste: 86.435%), demonstrating robust generalisation across diverse waste taxonomies and dataset scales.

**Table 12:** Accuracy comparison across algorithms and datasets.

Algorithm	HGCD	TrashNet	WasteNet	Realwaste
SMOBBCS	96.519	90.909	96.993	86.435
SMPSO	96.004	88.538	95.932	84.543
SMBAT	96.004	88.735	96.013	83.491
SMWOA	95.939	90.316	95.972	84.963
SMEPO	95.617	88.735	95.851	82.860
SMBCS	96.068	90.711	95.750	84.227
SMOBBCS Mean		92.71		
Best Rival Mean		91.80 (SMWOA)		

Green, best value per dataset; Mean = average across all four datasets.

**Table 13** presents Macro-F1 comparison across all algorithms and datasets. Similar to accuracy results, SMOBBCS achieves the highest Macro-F1 scores on all four datasets, with a mean of 0.9209 compared to best rival SMWOA’s mean of 0.9111. This consistent superiority on Macro-F1 — which addresses class imbalance by giving equal weight to all classes confirms that SMOBBCS’s gains represent genuine improvements in balanced minority-class detection rather than majority-class bias. **Table 14** presents accuracy vs. computational budget (population size × iterations) on the TrashNet dataset. Diminishing returns are observed above population size 30, justifying the choice of *N_p_* = 30 in all experiments.

**Table 13:** Macro-F1 comparison across algorithms and datasets.

Algorithm	HGCD	TrashNet	WasteNet	Realwaste
SMOBBCS	0.9538	0.8966	0.9693	0.8640
SMPSO	0.9450	0.8590	0.9588	0.8484
SMBAT	0.9449	0.8599	0.9594	0.8353
SMWOA	0.9454	0.8901	0.9592	0.8496
SMEPO	0.9398	0.8733	0.9579	0.8332
SMBCS	0.9472	0.8870	0.9568	0.8428
SMOBBCS Mean		0.9209		
Best Rival Mean		0.9111 (SMWOA)		

Green, best value per dataset; Mean, average across all four datasets.

**Table 14:** Accuracy (%) vs. computational budget (TrashNet dataset).

Pop\Iter	25	50	75	100
10	88.2	89.7	90.4	90.8
20	90.1	91.8	92.3	92.7
30	91.4	92.9	93.1	93.1
40	92.0	93.1	93.4	93.5
50	92.3	93.3	93.6	93.7

**Table 15** presents ablation study results on TrashNet with CNN-DNN architecture, comparing SMOBBCS against Cuckoo-Only (CS-Only), Bat-Only (BA-Only), fixed-parameter baseline, random search, and grid search variants. SMOBBCS outperforms CS-Only by +3.4% and BA-Only by +2.7%, demonstrating the synergistic hybrid advantage of the combined bat-cuckoo mechanism.

**Table 15:** Ablation study results (TrashNet, CNN-DNN).

Variant	Acc (%)	Std	F1-Mac	HV	Spacing	Iter to 90%
**SMOBBCS**	**93.1**	0.8	**0.926**	**0.856**	**0.092**	**42**
CS-Only	89.7	1.2	0.891	0.801	0.134	58
BA-Only	90.4	1.1	0.898	0.817	0.118	51
Fixed-Params	91.2	1.0	0.906	0.829	0.105	48
Random-Search	87.3	2.1	0.867	0.764	0.187	N/A
Grid-Search	89.7	0.3	0.891	0.798	0.142	N/A

Green, best value per metric.

### 5.8 Limitations and future work

Despite its strong empirical performance, SMOBBCS is subject to several limitations. All datasets are primarily from controlled or semi-controlled environments with limited geographic diversity; real sorting facilities encounter occlusions, motion blur, variable lighting, and contamination not fully represented in benchmark datasets. Moreover, category taxonomy inconsistencies across datasets (e.g., “plastic” vs. “hard plastic” vs. “PET”) complicate cross-dataset transfer learning. Full optimisation also requires substantial GPU-hours per dataset depending on architecture. Each candidate evaluation requires full model training, making population sizes above 50 exhibit diminishing returns. Additionally, SMOBBCS optimises three continuous hyperparameters but not the neural architecture itself (the ResNet18 backbone is fixed). The bi-objective formulation also omits inference latency, model size, and energy consumption as objectives. Finally, despite the hybrid approach, convergence to the true Pareto-optimal front cannot be theoretically guaranteed. A proposed multi-fidelity successive-halving scheme evaluating all candidates on 25% of the training data for 10 epochs, retaining the top 50% for training on 50% of the data, and retaining the top 25% for full training could achieve faster speedups. Extending the formulation from two objectives to five (accuracy, F1, inference latency, model size, and energy consumption) would likely require parallel-coordinates visualisation and hypervolume-based selection. Future work could also explore end-to-end waste-sorting robot integration, combining the SMOBBCS-optimised classifier with reinforcement-learning-based grasping and multi-bin placement.

## 6 Conclusion

In an era of rapid urbanisation, smart cities require efficient waste management to maintain clean and green environments. A critical challenge today is the accurate classification of garbage types; correct implementation can result in environmental conservation and more efficient recycling processes. However, classification has predominantly relied on human supervision, which introduces high error rates and environmental risks. This study investigates the automation of this process using deep learning and evolutionary computation, specifically the Surrogate-Assisted Multi-Objective Binary Bat-Cuckoo Search algorithm (SMOBBCS), which is designed to address the dual challenge of maximising waste-classification accuracy whilst preserving balanced minority-class detection across highly imbalanced waste categories. The algorithm applies a time-varying adaptive gate that executes a disciplined transition from bat-style frequency-modulated exploitation in early search phases to Lévy-flight-driven global exploration, and is accelerated by a *k*-NN surrogate model that reduces true deep-learning evaluations by 65% per iteration without measurable degradation in Pareto archive quality. Extensive empirical validation across four heterogeneous real-world waste benchmarks (HGCD, TrashNet, WasteNet, and Realwaste), evaluated with four deep-learning classifiers (CNN, DNN, GRU, and CNN-DNN), demonstrated consistent, quantifiable improvements. SMOBBCS achieved mean accuracy of 92.71%, mean Macro-F1 of 0.9209, mean precision of 0.9225, mean recall of 0.9205, and mean MCC of 0.9052, outperforming all five surrogate-assisted multi-objective competitors (SMEPO, SMWOA, SMBCS, SMPSO, SMBAT) across all metrics. Statistical validation test confirms that SMOBBCS improvements are neither random fluctuations nor dataset-specific artefacts, but systematic advantages generalising across diverse waste classification scenarios. Furthermore, the synergistic combination of bat exploitation and cuckoo exploration within a coherent multi-objective surrogate framework constitutes a compelling, practically deployable approach to hyperparameter optimisation for modern, large-scale waste classification systems. As waste generation continues its exponential growth trajectory, automated classification systems optimised by algorithms such as SMOBBCS can play an increasingly critical role in achieving circular economy goals and enabling sustainable resource management at scale.

## Data Availability

The original contributions presented in the study are included in the article/Supplementary Material. Further inquiries can be directed to the corresponding author.
